# Combined Metabolomics and Network Pharmacology Analysis Reveal the Effect of Rootstocks on Anthocyanins, Lipids, and Potential Pharmacological Ingredients of Tarroco Blood Orange (*Citrus sinensis* L. Osbeck)

**DOI:** 10.3390/plants13162259

**Published:** 2024-08-14

**Authors:** Lei Yang, Shuang Li, Yang Chen, Min Wang, Jianjun Yu, Wenqin Bai, Lin Hong

**Affiliations:** 1 Fruit Tree Research Institute, Chongqing Academy of Agricultural Sciences, Chongqing 401329, China; leir8512@126.com (L.Y.); sclishuang61@163.com (S.L.); wm950918@126.com (M.W.); jerksion@163.com (J.Y.); 2 Key Laboratory of Evaluation and Utilization for Special Crops Germplasm Resource in the Southwest Mountains, Ministry of Agriculture and Rural Affairs, Chongqing 401329, China; lixianchy2008@163.com; 3 Biotechnology Research Institute, Chongqing Academy of Agricultural Sciences, Chongqing 401329, China

**Keywords:** tarocco, rootstocks, metabolomics, antioxidant mechanisms, anthocyanin

## Abstract

The benefits of citrus fruits are strongly associated with their secondary metabolites. In this study, we conducted widely targeted metabolomics analyses to compare the variability of the ingredients in four scion–rootstock combinations. A total of 376 differential metabolites were obtained by a multivariate statistical analysis, and a KEGG pathway analysis showed that the enriched metabolic pathways were mainly related to the biosynthesis of flavonoids as well as lipid metabolism. The anthocyanin-targeted metabolomic features showed that cyanidin 3-O-glucoside, cyanidin 3-O-(6-O-malonyl-beta-D-glucoside), cyanidin 3-O-sophoroside, and cyanidin 3-O-xyloside were the pigments responsible for the red color of Tarocco. A lipid metabolomics analysis revealed that when Tarocco was hetero-grafted with rootstock H, there was an increase in the content of each lipid subclass, accompanied by an increase in the levels of unsaturated fatty acids, including polyunsaturated linoleic and linolenic acids, thus impacting the ratio of unsaturated fatty acids to saturated fatty acids. Additionally, we determined their antioxidant capacity (‘Trifoliate orange’ (Z) > ‘Citrange’ (ZC) > ‘Hongju’ (H) > ‘Ziyang Xiangcheng’ (X)) using in vitro assays. Finally, we utilized a network pharmacology analysis to explore the antioxidant mechanisms and potential pharmacological ingredients; we obtained 26 core targets proteins and 42 core metabolites associated with oxidative damage, providing a basis for future preventive and therapeutic applications of these metabolites.

## 1. Introduction

As a result of their healthy qualities, blood oranges (*Citrus sinensis* L. Osbeck) are becoming more popular. Citrus, including blood orange, is a natural source of many antioxidants and bioactive substances, rich not only in primary metabolites, but also in valuable secondary metabolites [1,2]. Notably, citrus fruits contain a large number of phenolic acids such as ferulic acid, *p*-coumaric acid, sinapic acid, caffeic acid, and sinapic acid, which possess robust antioxidant activity [3,4]. They also contribute to the formation of the fruit’s color, bitterness, astringency, antioxidant activity, and flavor [5]. Therefore, phenolic compounds play an important role in the organoleptic quality of citrus [6]. Similarly, in addition to their beneficial health properties, flavonoids are natural compounds. These compounds exhibit several biological properties, including antioxidant and anticancer effects [7]. Flavonoids are one of several possible cancer prevention agents [8]. Hesperidin and diosmin have been documented to exhibit a wide range of pharmacological properties, including anti-inflammatory, anticarcinogenic, and hypolipidemic activities [9]. For quercitrin, genistein (genistin), and isoscopoletin, there are similar research reports indicating their activity [10,11]. Anthocyanins are the most common pigment compounds, and belong to a large group of phenolic secondary metabolites known as flavonoids, which contribute to the distinctive red color of blood oranges, and are also associated with human health benefits as antioxidants [12,13,14]. Research has demonstrated that monounsaturated fatty acids and certain polyunsaturated fatty acids, such as oleic acid, linoleic acid, and linolenic acid, have significant impacts on the prevention and inhibition of cardiovascular diseases [3,15,16]. Additionally, synephrine, an alkaloid compound, has been shown to improve alloxan-induced diabetes mellitus by inhibiting oxidative stress [17].

However, the distribution and content of these compounds are not uniform in the fruit at different stages of development. Grafting is a time-honored and traditional horticultural technique employed to enhance crop qualities [18,19,20]. Studies have demonstrated that grafting can influence the biosynthesis of secondary metabolites, leading to variations in metabolite compositions between grafted and non-grafted plants [21,22,23]. Different rootstocks, citrus varieties, and even different strains of rootstock ear combinations exhibit significant differences, ultimately affecting their chemical compositions [1]. Therefore, rootstock selection is important in citrus cultivation, as it affects tree potential and fruit quality. For decades, ‘Ziyang Xiangcheng’ (*C. junos* Sieb. ex Tanaka), ‘Hongju’ (*C. reticulata* Blanco), ‘Trifoliate orange’ (*Poncirus trifoliata* L. Raf.), and ‘Citrange’ (*Poncirus trifoliate* L. raf. x *Citrus sinensis* L. Osbeck.) have remained the most commonly cultivated varieties in Sichuan Province, as well as in other key citrus-growing regions across China.

In recent years, network pharmacology has emerged as an effective strategy to analyze the potential therapeutic targets of drug interventions and disease [24,25,26,27]. This method combines diverse fields such as pharmacology, statistics, and omics to perform network analysis on drug components and disease targets [28]. Network pharmacology analysis has the potential to uncover the mechanisms behind various actions, providing fresh perspectives for research on plant materials. For instance, researchers employed both network pharmacology and ABTS free radical scavenging activity techniques to reveal the antioxidant mechanisms of potentially active compounds in mung bean [29]. Additionally, this approach has quickly become useful for predicting ingredient targets and discovering potential pharmacological components and new drugs, thereby offering valuable tools for the advancement and utilization of natural plant materials.

Metabolomics approaches have proven to be essential, merging analytical methods with computational and statistical analyses [30]. This integration has significantly sped up the screening and identification of active compounds [31]. Despite recent advancements in the application of metabolomics to study rootstock-mediated effects on secondary metabolites in various plants, including citrus, grapevine, and apple, our understanding of how rootstocks affect citrus fruit development is still limited [15,21,32,33,34].

In this study, we extensively analyzed the differences and similarities in metabolites among four scion–rootstock combinations, namely, Z, ZC, H, and X, utilizing a widely targeted metabolomics approach combined with anthocyanin- and lipid-targeted metabolomics. Additionally, we compared the antioxidant activity of four scion–rootstock combinations and elucidated the underlying mechanisms by combining metabolomics and network pharmacology. This study could enhance our knowledge of the active ingredients and health benefits of citrus, as well as provide a theoretical foundation for their further development and utilization.

## 2. Results

### 2.1. Effect of Grafting on Fruit Flavor, Nutritional Quality, and Antioxidant Capacity

**Figure 1:**
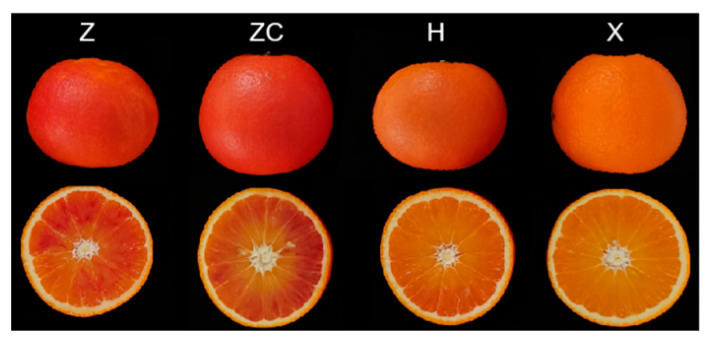
The phenotype of Tarocco fruits grafted with four different rootstocks.

In this study, the widely cultivated variety Tarocco was utilized and hetero-grafted onto four rootstocks, namely, Z, ZC, H, and X. Each scion–rootstock combination of citrus fruits was harvested at the ripening stage. As depicted in Figure 1, it is evident that fruits from rootstocks Z and ZC exhibit a deeper red coloration compared with H and X. Furthermore, the fruit flavor and nutritional quality of Tarocco were significantly influenced by the rootstock (Table 1). Compared with H and X, rootstocks Z and ZC significantly increased the content of fruit flavor compounds, including the total soluble solids (TSS), TSS/TA, and the content of glucose, sucrose, and fructose. Additionally, Z and ZC also resulted in a notable elevation in the content of total phenolic acids (TPC), total flavonoids (TFC), and total anthocyanins (TAC), but did not affect the levels of vitamin C and total amino acids, compared with H and X.

Citrus is a natural source of numerous antioxidants and bioactive substances, such as flavonoids, anthocyanins, and phenolic acids. These compounds are closely associated with the antioxidant capabilities of citrus fruits. In this study, FRAP, ABTS, and DPPH methods were employed to determine the total antioxidant capacity of blood orange juice grafted with different rootstocks. As shown in Table 1, rootstock Z and ZC exhibited a significantly elevated antioxidant capacity compared with H and X. A correlation analysis revealed strong correlations between the TPC and antioxidant indexes, with coefficients of 0.91 for FRAP, 0.90 for ABTS, and 0.88 for DPPH, as well as coefficients of TFC and TAC exceeding 0.89. These findings highlight a positive relationship between the total flavonoid and phenolic acid levels and antioxidant capacity. Additionally, our research revealed a significant association between the TPC and TFC, with a high correlation coefficient of 0.97 in citrus fruit extracts (Table S1).

### 2.2. Secondary Metabolic Profiling of Four Scion–Rootstock Combinations

**Figure 2:**
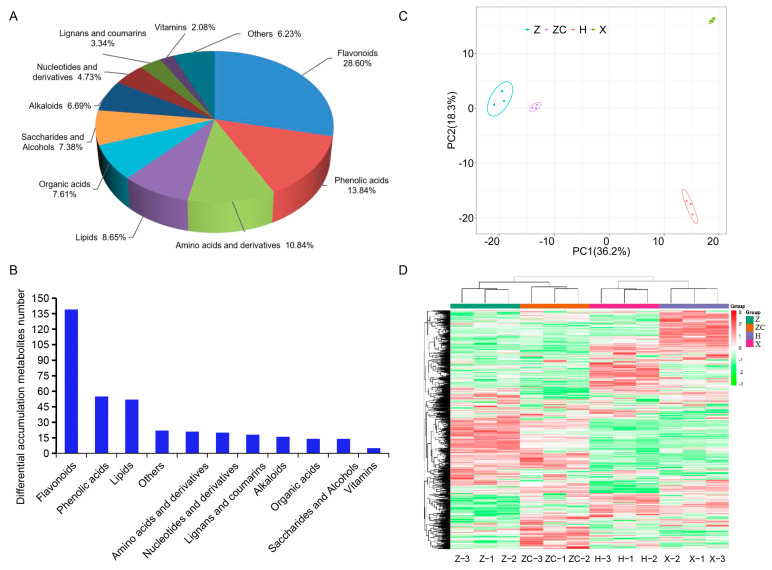
Metabolic profile of Tarocco blood orange on four rootstocks (Z, ZC, H, and X). (**A**) Classification of the 867 secondary metabolites of four scion–rootstock combinations. (**B**) Differential landscape of metabolites in Tarocco blood orange with four rootstocks. (**C**) PCA score plots. (**D**) HCA of secondary metabolites.

To investigate the variations in the metabolite content and composition during citrus fruit development, we carried out a widely targeted metabolite method based on UPLC-MS/MS to analyze four scion–rootstock combinations. A total of 867 metabolites were detected in four fruit samples, which could be classified into eleven different categories; flavonoids, phenolic acids, and amino acids and derivatives are the most three abundant compounds (Figure 2A, Table S2). We used OPLS-DA to screen out the differential metabolites between pairwise comparisons of Z, ZC, H, and X (Figures S1 and S2). Pairwise comparisons between the samples revealed 376 metabolites with differential expression (Table S3). Notably, among all the differential metabolites, the flavonoids showed the greatest variability, followed by phenolic acids and lipids (Figure 2B). The KEGG pathway analysis showed that the enriched metabolic pathways were mainly related to the biosynthesis of flavonoids, including “anthocyanin biosynthesis”, “flavone and flavonol biosynthesis”, “isoflavonoid biosynthesis”, and “flavonoid biosynthesis”, as well as lipid metabolism, including “glycerophospholipid metabolism”, “fatty acid biosynthesis”, “ether lipid metabolism”, “linoleic and linolenic acid biosynthesis”, and “linolenic acid metabolism” (Figure S3). The validity of the data was confirmed by a principal component analysis (PCA), which clearly distinguished the samples from different groups (Figure 2C). Additionally, the heat map from the Hierarchical Cluster Analysis (HCA) distinctly showed that these secondary metabolites could be categorized into four distinct subgroups (Figure 2D). Aligning with the PCA results, the data revealed significant differences in the secondary metabolite profiles among the groups.

### 2.3. Targeted Metabolomic Analyses Detected Metabolites Related to Anthocyanin Synthesis

**Figure 3:**
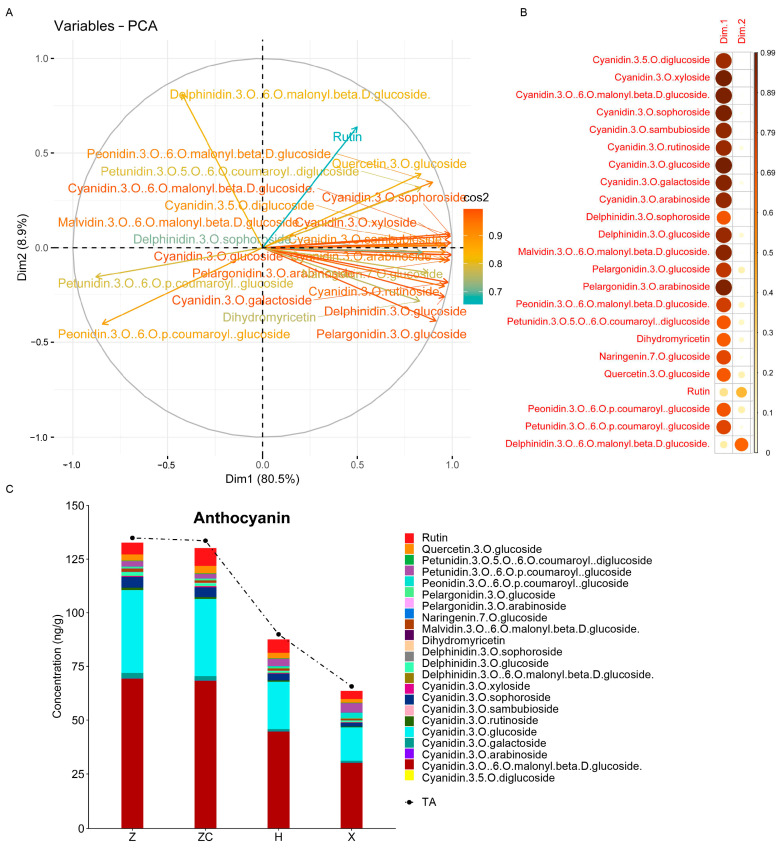
Unsupervised multivariate PCA analyses of metabolites and their association with pulp color. (**A**) In the variable correlation plots of 44 metabolites, the distances between variables and the origin measure the quality of the variables on the factor map colored by cos2 value for PC1 and PC2. (**B**) A heatmap of cos2 values of variables in two dimensions. (**C**) The concentration of 23 DAMs (stacked bar chart) and total anthocyanins (dotted line) in Tarocco fruit samples from four different rootstocks.

According to previous studies, the reason why Z and ZC displayed more pronounced coloration compared with H and X is mainly because of the difference in anthocyanins. Therefore, targeted metabolomics was employed to investigate the difference in anthocyanins among the four scion–rootstock combinations. In total, 44 types of metabolites related to anthocyanins were identified and classified into seven groups: cyanidin, peonidin, delphinidin, malvidin, pelargonidin, petunidin, and others (Table S4). An unsupervised multivariate PCA showed that the first three principal components explained 81.8% of the variance. Notably, PC1 and PC2, which represented 51.1% and 17.0% of the variance, respectively, effectively described the distribution of compounds in the samples (Figure 3A). Fourteen compounds, predominantly colored anthocyanins, were explained better in PC1. These compounds included nine cyanidins, two pelargonidins (pelargonidin 3-O-arabinoside and pelargonidin 3-O-glucoside), peonidin 3-O-(6-O-malonyl-beta-D-glucoside), delphinidin 3-O-glucoside, and malvidin 3-O-(6-O-malonyl-beta-D-glucoside). However, delphinidin 3-O-galactoside and petunidin 3-O-sambubioside demonstrated a more pronounced association with PC2 (Figure 3B, and Table S5, variable correlation >0.8). In particular, cyanidin, which comprised 74.7–86.9% of the anthocyanin content in all four scion–rootstock combinations, demonstrated higher concentrations in the fruit samples from rootstocks Z and ZC in comparison to those from rootstocks H and X (Table S6). Moreover, both cyanidin 3-O-glucoside and cyanidin 3-O-(6-O-malonyl-beta-D-glucoside) consistently appeared as the two most abundant anthocyanins across all four scion–rootstock combinations, constituting a proportion that ranged from 69.5% to 80.0% of the total anthocyanin content (Figure 3C, Table S6). The levels of 23 metabolites, including 9 cyanidins, were significantly higher in the fruit samples from rootstocks Z and ZC than in those from rootstocks H and X (Figure 3C and Table S6). Taken together, the interplay between the phenotype and metabolomics highlighted a critical period associated with grafting.

### 2.4. Targeted Lipidomics Analyses Detected Metabolites Related to Lipid Synthesis

**Figure 4:**
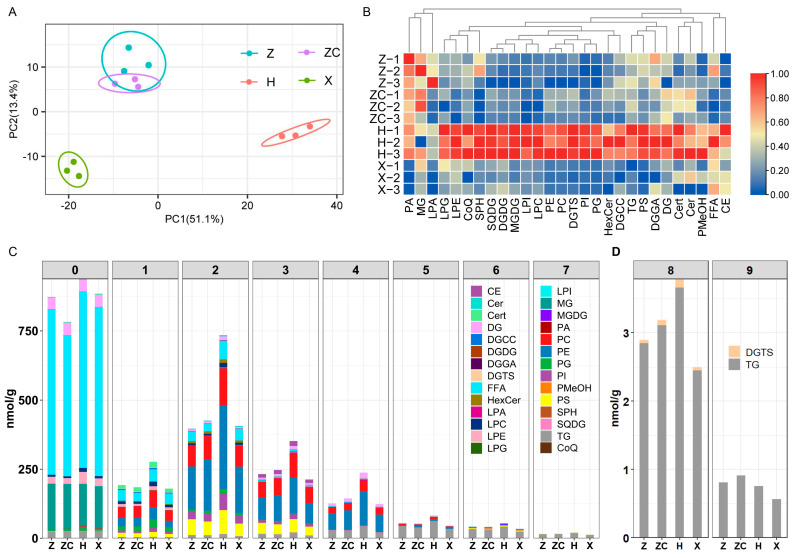
Lipidomics profile of Tarocco blood orange from four rootstocks (Z, ZC, H, and X). (**A**) PCA analyses of lipids. (**B**) Heatmap of each lipid subclass concentration. (**C**,**D**) The unsaturation of FFAs and fatty acids on the acyl chain in fruit samples from four rootstocks.

A total of 747 lipid species from 28 subclasses were analyzed in the four treatment groups (Table S7). These included extraplastidic phospholipids (PE, PC, PI, PA, PG, and PS), lysolipids (LPA, LPC, LPI, LPG, and LPE), glycerolipids (MG, DG, and TGs), glucosylsphingoshine (plastidic lipids) (DGMG, DGDG, MGDG, and SQDG), sphingoid (Cer, HexCer, PhytoSph, and SPH), free fatty acids (FFAs), prenol lipids (CoQ), and others. The results of the unsupervised PCA indicated a noticeable distinction in the samples from rootstock H when compared to the other three samples (Figure 4A). Furthermore, the total content of each subclass from rootstock H was generally higher than that from the other three rootstocks (Figure 4B and Table S8). These findings were consistent with the data obtained from the widely targeted metabolomics analysis. Additionally, the unsaturation levels of FFAs and fatty acids within the acyl chain of the fruit samples from rootstock H were found to be higher than those from the other three rootstocks (Figure 4C and Table S9).

A total of 241 differentially accumulated lipids were identified at the intersection of three metabolite sets (Z vs. H, ZC vs. H, X vs. H). These lipids included PC, PE, DGMG, DGDG, MGDG, SQDG, and TG. Remarkably, their concentrations were found to be the highest in the Tarocco hetero-grafted with rootstock H (Figure S4, Table S10). Moreover, the Tarocco hetero-grafted with H exhibited elevated levels of polyunsaturated linoleic and linolenic acids, along with a higher ratio of unsaturated fatty acids to saturated fatty acids (UFA/SFA) in the fruit samples, compared to those of the other three rootstocks (Figure S5). These results collectively highlight that grafting Tarocco onto rootstock H significantly enhances both the concentration and unsaturation of lipids.

### 2.5. Network Pharmacology Analysis

**Figure 5:**
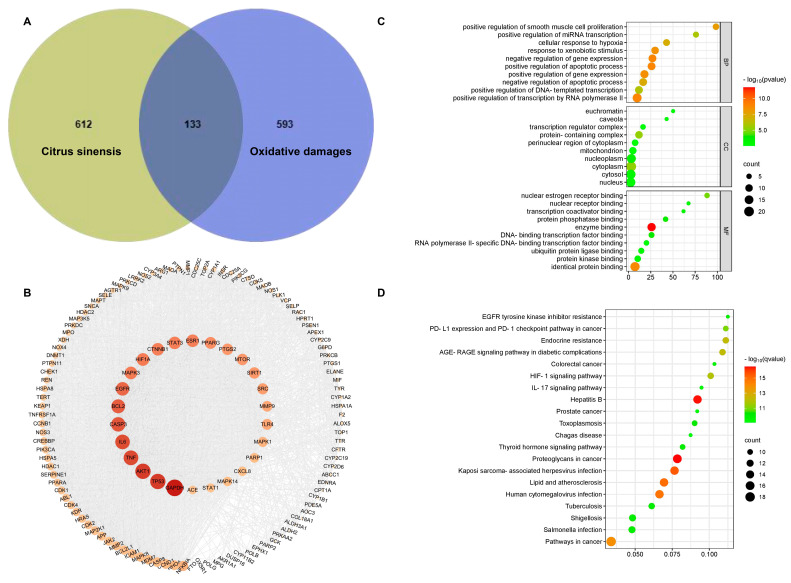
Network pharmacology analysis. (**A**) Venn diagram of overlapping targets between citrus and oxidative damage; the overlapping targets represent antioxidant activity-related proteins targeted by potentially active ingredients identified in citrus. (**B**) Core target network diagram analyzed by Cytoscape using the overlapping target data. The target near the center of the circle is the core target. (**C**) GO enrichment analysis of the core target. The top 10 GO functional terms were shown. (**D**) KEGG pathway enrichment analysis of the core target. The top 20 pathways were shown.

To investigate the potential antioxidant mechanism further, we conducted a network pharmacology analysis. Finally, we successfully identified a total of 76 ingredients (Table S11). Among these, we screened 58 potentially active ingredients linked to 726 protein targets (Table S12). Additionally, we searched a total of 745 targets of oxidative damage in the OMIM, DisGeNET, and GeneCards databases (Table S12). Notably, 133 common targets shared between citrus targets and oxidative damage were identified (Figure 5A, Table S12). Subsequently, these common targets were assessed using the SRING database and Cytoscape, and 26 core targets were identified (Figure 5B, Table S12). This result showed that citrus fruits primarily mitigate oxidative damage by regulating the expression levels of *GAPDH*, *TP53*, *AKT1*, *MAPKs*, and other genes, providing valuable insights into their potential therapeutic applications (Table S12).

To further understand the mechanisms underlying the antioxidant activity of citrus, we conducted a GO functional and KEGG pathway enrichment analysis on the 26 core target genes (Figure 5). The GO functional analysis showed that the biological processes (BPs) primarily encompassed apoptotic processes, gene expression, and so on. Regarding the cellular components (CCs), the notable entries included cytoplasm, nucleus, cytosol, and so on. In terms of the molecular function (MFs), the enriched terms mainly involved binding to identical proteins, enzymes, and so on (Figure 5C). These results effectively demonstrated that citrus alleviated oxidative stress damage by modulating biological processes in multiple cellular compartments and functions. Notably, the oxidative damage-related targets analyzed by the KEGG pathway enrichment were mainly involved in signaling pathways (HIF-1, IL-17, Thyroid hormone, and AGE-RAGE), cancer pathways (proteoglycans in cancer, PD-L1 expression and PD-1 checkpoint pathway in cancer, prostate cancer), herpesvirus and bacterial infection (kaposi sarcoma-associated herpesvirus infection, Human cytomegalovirus infection, salmonella infection), hepatitis B, lipid and atherosclerosis, and so on (Figure 5D). These results indicated that the antioxidants in citrus exert their antioxidant effects by affecting pathways involved in cancer and pathogen infection. Therefore, the utilization of citrus holds significant promise in the prevention and treatment of these diseases.

### 2.6. The Effect of Four Rootstocks on Core Metabolites

**Figure 6:**
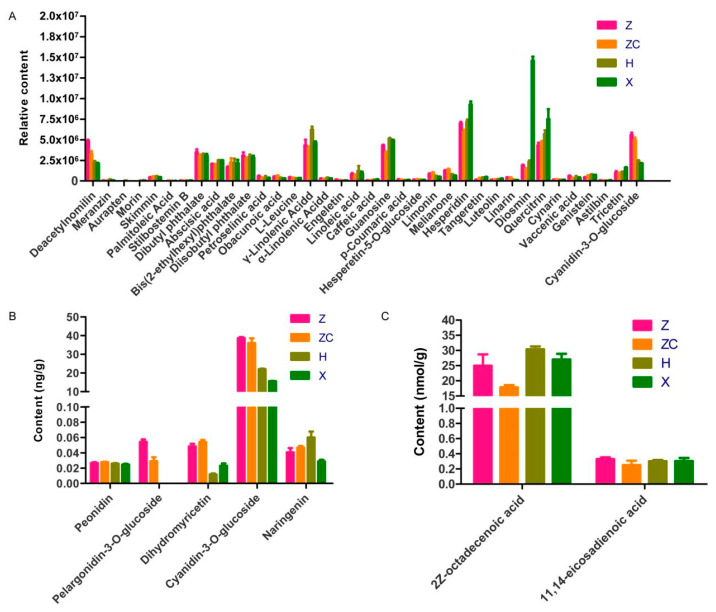
(**A**) Relative content of core metabolites from widely targeted metabolomics data. Relative content was calculated by calculating the peak area formed by the characteristic ions of each substance in the detector. (**B**) Content of core metabolites from anthocyanin-targeted metabolomics data. (**C**) Content of core metabolites from lipid-targeted metabolomics data.

Based on the core targets identified above, we found 42 core metabolites associated with oxidative damage (Figure S6A). These compounds can be divided into nine classes, the top three being flavonoids (42%), free fatty acids (20%), and phenolic acids (15%) (Figure S6B). Their content varied in different scion–rootstock combinations (Figure 6, Table S13). Eleven core metabolites (deacetylnomilin, cyanidin-3-O-glucoside, diisobutyl phthalate, dibutyl phthalate, bis (2-ethylhexyl) phthalate, abscisic acid, guanosine, γ-linolenic Acid, quercitrin, hesperidin, diosmin) were found to have a higher content in four scion–rootstock combinations as determined through the widely targeted metabolomics analysis (Figure 6A). Specifically, the metabolites diosmin, quercitrin, and hesperidin exhibited the highest content in scion–rootstock combination X, while cyanidin-3-O-glucoside and deacetylnomilin had a higher content in combinations Z and ZC compared to combinations H and X. Additionally, γ-linolenic acid had the highest content in combination H compared to the other combinations. Furthermore, the content of cyanidin-3-O-glucoside as determined through anthocyanin-targeted metabolomics data was consistent with that found through the widely targeted metabolomics analysis, with combinations Z and ZC showing a higher content compared to combinations H and X (Figure 6B). The content of 2Z-octadecenoic acid as determined through lipid-targeted metabolomics data had the highest content in combination H compared to the other combinations (Figure 6C).

## 3. Discussion

### 3.1. Rootstock Z and ZC Could Promote Anthocyanin Accumulation

Blood oranges (*Citrus sinensis* L. Osbeck) are sweet orange cultivars with red-fleshed fruit, and they are characterized by a high content of anthocyanins in their edible portions, which sets them apart from blond varieties [12]. Tarocco is one of the most common and widespread blood orange varieties in the Mediterranean climate area [35]. The rootstock used for grafting the scion cultivars significantly influences citrus production [20,36,37]. Several studies have shown that grafting can influence the chemical composition of citrus during its development [20,36,37]. However, our understanding of the effects of rootstocks on citrus fruit during development remains limited. In this study, we focused on Tarocco, a widely cultivated citrus variety, and investigated its performance when hetero-grafted onto four different rootstocks, namely, Z, ZC, H, and X. Our analysis of fruit samples from rootstocks Z and ZC revealed enhanced coloration compared to those from rootstocks H and X, indicating the significant influence of the rootstock on fruit quality (Figure 1 and Table 1). A subsequent analysis delved into the metabolite content and composition, employing a widely targeted metabolomics approach. The pairwise comparisons exposed 376 metabolites with differential expression (Table S2 and Figure S1B). A KEGG pathway analysis highlighted enrichments in metabolic pathways predominantly associated with flavonoid biosynthesis and lipid metabolism (Figure S2). These findings underscore the significant impact of grafting Tarocco onto specific rootstocks on metabolite profiles.

A further exploration of anthocyanins using anthocyanin-targeted metabolomics uncovered a positive correlation between most anthocyanins and fruit coloring (Figure 3A). Previous studies proved that pelargonidin-, cyanidin-, and delphinidin-based anthocyanins play a role in determining colors such as brick red/scarlet, red/magenta, and violet/blue, respectively [38]. Specifically, cyanidin, the most abundant anthocyanin in all the scion–rootstock combinations, displayed increased concentrations in the fruit samples from rootstocks Z and ZC compared with those from H and X (Figure 3C and Table S3). The pigments responsible for the red color of the Tarocco cultivar included cyanidin 3-O-glucoside, cyanidin 3-O-(6-O-malonyl-beta-D-glucoside), cyanidin 3-O-sophoroside, and cyanidin 3-O-xyloside (Figure 3 and Table S3). Remarkably, cyanidin 3-O-glucoside and cyanidin 3-O-(6-O-malonyl-beta-D-glucoside) were the predominant constituents among the anthocyanins, with higher concentrations observed in the fruit samples obtained from rootstocks Z and ZC compared with H and X (Figure 3C and Table S4). This observation highlights a robust association between grafting onto specific rootstocks and anthocyanin accumulation, likely due to the significant role rootstocks play in the synthesis and accumulation of various anthocyanin types [39,40].

### 3.2. Rootstock H Could Promote UFA Synthesis

It is widely acknowledged that incorporating a diet rich in unsaturated fatty acids as part of a healthy eating regimen shows great potential in the prevention and management of cardiometabolic diseases [41]. Our findings indicated significantly elevated levels of unsaturation in both free fatty acids and fatty acids within the acyl chain in rootstock H (Figure 4C and Table S8), along with a higher UFA/SFA ratio in the fruit samples (Figure S5C). PUFAs, known for their bioactive properties, have been widely recognized for their potential benefits in combating cardiovascular disease, cancer, oxidative stress, and inflammation owing to its antioxidant and anti-inflammatory properties [42,43,44]. Our study revealed that Tarocco grafted onto rootstock H exhibited elevated levels of polyunsaturated linoleic and linolenic acids compared to counterparts grafted onto other rootstocks (Figure S5A,B). These findings suggest that rootstock H may serve as a promising avenue for enhancing the presence of UFAs, including PUFAs, known for their advantageous effects on human health.

### 3.3. Rootstocks Z and ZC Could Promote Antioxidant Activity by Increasing TPC, TFC, and TAC Content

Phenolic compounds, including anthocyanins, are considered non-enzymatic antioxidant compounds because they act as electron donors, contributing to the neutralization of free radicals [45,46,47]. In vitro studies have demonstrated the high antioxidant capacity of orange juices due to their phenolic contents, which include flavonoids and phenolic acids [48,49]. Consistent with previous studies, our results show that the total phenolic content, total flavonoid content, and total anthocyanin content are positively correlated with the antioxidant capacity (Table 1). Although the antioxidant potential of citrus species has been extensively investigated [50,51], there have been few reports on the effect of rootstocks on the antioxidant activity of blood oranges [52,53]. Antioxidant capacity assessments have revealed that rootstocks Z and ZC enhance the antioxidant activity of Tarocco blood oranges compared to the other two rootstocks (Figure S4). Furthermore, the TPC is significantly related to the TFC (Table 1), which is reasonable since the biosynthesis of many flavonoids occurs downstream of phenolic acids in plants [54,55]. Taking all the above analyses into account, compared with the scion–rootstock combinations H and X, combinations Z and ZC exhibited a higher TPC, TFC, and TAC, which positively correlated with the antioxidant activity.

### 3.4. Network Pharmacology Analysis Reveals Antioxidant Mechanism of Blood Orange

TCMSP is a prominent platform for the systemic pharmacology of herbal medicines, allowing researchers to explore the connections between drug components, targets, and diseases [56]. Through our extensive analysis, we identified 76 ingredients in total (Table S11). Out of these, 58 potentially active ingredients were selected, linked to 726 protein targets listed in the TCMSP database (Table S12). Furthermore, we identified 745 targets related to oxidative damage using the OMIM, DisGeNET, and GeneCards databases (Table S12). We discovered 133 common targets shared by citrus and oxidative damage (Figure 5A, Table S8). The STRING and Cytoscape analyses revealed 25 core targets that are integral to various interconnected reactions associated with oxidative stress (Figure 5B, Table S12). Previous research indicates that Mitogen-activated protein kinases (MAPKs) and the epidermal growth factor receptor (EGFR) are crucial in antioxidant, anti-aging, and antitumor immune responses [57,58]. These discoveries offer valuable insights into the potential therapeutic uses of citrus. The GO functional and KEGG pathway enrichment analyses of the 26 core target genes showed that citrus mitigates oxidative stress damage by influencing biological processes in various cellular compartments, affecting pathways linked to cancer and cardiovascular diseases. For instance, limonium aureum extract has demonstrated antioxidant effects by modulating the activities of antioxidant enzymes and lipid peroxidases in H_2_O_2_-induced RAW264.7 cells [59]. HIF-1 is considered a key mediator in shifting carbohydrate metabolism from oxidative phosphorylation to accelerated glycolysis, associated with the antioxidant and anti-radiation properties of cancer cells [60]. Therefore, citrus holds considerable potential for preventing and treating these diseases.

Based on the core targets identified above, we found 40 core metabolites associated with oxidative damage (Figure S6A). These compounds can be divided into nine classes, the top three being flavonoids (42%), free fatty acids (20%), and phenolic acids (15%) (Figure S6B). Their content varied in the different scion–rootstock combinations (Figure 6, Table S13). Eleven core metabolites (Deacetylnomilin, Cyanidin-3-O-glucoside, Diisobutyl phthalate, Dibutyl phthalate, Bis (2-ethylhexyl) phthalate, abscisic acid, guanosine, γ-linolenic acid, quercitrin, hesperidin, diosmin) were found to have a higher content in four scion–rootstock combinations as determined through widely targeted metabolomics analysis. Specifically, the metabolites diosmin, quercitrin, and hesperidin exhibited the highest content in scion–rootstock combination X, while cyanidin-3-O-glucoside and deacetylnomilin had higher content in combinations Z and ZC compared to combinations H and X. Additionally, γ-linolenic acid had the highest content in combination H compared to the other combinations. Furthermore, the content of cyanidin-3-O-glucoside as determined through targeted metabolomics data was consistent with that found through the widely targeted metabolomics analysis, with combinations Z and ZC showing a higher content compared to combinations H and X. These results indicated that four rootstocks have different effects on the content of core compounds. These findings provide valuable insights into the selection of rootstocks to optimize the production of specific compounds.

## 4. Conclusions

This study showed that the rootstock Z and ZC accelerated the accumulation of anthocyanins concomitant with an enhancement of antioxidant capacity, while rootstock H had a greater effect on the lipid concentration and composition. Three pigments, namely, cyanidin 3-O-glucoside, cyanidin 3-O-sophoroside, cyanidin 3-O-(6-O-malonyl-beta-D-glucoside), and cyanidin 3-O-xyloside, were responsible for the red coloration of Tarocco fruits. Notably, cyanidin 3-O-glucoside and cyanidin 3-O-(6-O-malonyl-beta-D-glucoside) emerged as the predominant constituents among the anthocyanins, displaying elevated concentrations in fruit samples obtained from rootstocks Z and ZC. A lipidomic analysis showed that when Tarocco was hetero-grafted with rootstock H, there was an increase in the unsaturation levels of free fatty acids, including polyunsaturated linoleic and linolenic acids, which have significant impacts on the prevention and inhibition of cardiovascular diseases. A network pharmacology analysis revealed that the antioxidative mechanism of citrus may be related to the MAPK, EGFR, and HIF-1 signaling pathways. Finally, we obtained 26 core target proteins and 42 core metabolites associated with oxidative damage, providing a basis for future preventive and therapeutic applications of these metabolites. These findings provide valuable insights for improving the application value of grafting by enhancing the accumulation of anthocyanins and lipid components in blood orange plants.

## 5. Materials and Methods

### 5.1. Plant Materials and Sample Collection

The tarocco blood orange variety was grafted onto four rootstocks: ‘Trifoliate orange’ (*Poncirus trifoliata* L. Raf), ‘Carrizo citrange’ (*Citrus sinensis* Osb. × *P. trifoliate* Raf), ‘Hongju’ (*Citrus reticulata* Blanco cv. Red tangerine), and ‘Ziyang Xiangcheng’ (*Citrus junos* Sieb. ex Tanaka). These four rootstock samples are hereinafter referred to as Z, ZC, H, and X, respectively. At the ripening stage, fruit samples were collected from nine blood orange trees with the same age (6 years) and similar fruit-bearing capacity. These trees represented four different rootstocks. Three sample trees were randomly selected from each treatment, and five fruits were picked from the four directions of each sample tree. A total of 15 fruits with average size and similar color were selected as samples. Each treatment was repeated 3 times, there were 3 sample trees in each replication.

### 5.2. Fruit Quality and Major Functional Nutrients Determination

The total soluble solids (TSS) was determined using a digital refractometer (PAL-1; Atago, Tokyo, Japan). Titratable acids (TA) were determined by acid-base titration. The vitamin C (Vc) content was determined by titration with 2,6-dichloroindophenol. The contents of glucose (GC), sucrose (SC), fructose (FC), total anthocyanins (TAC), and total amino acids (TAA) were determined by Norminkoda Biotechnology Co., Ltd., Wuhan, China.

The fresh fruit samples were ground to homogenize them in liquid nitrogen. Subsequently, 1 g of the powdered samples was subjected to ultrasound extraction (Ultrasound Frequency: 40 kHz, Ultrasound Power: 300 watts) for 60 min at 40 °C using 10 mL of 80% methanol. After this extraction, the supernatant was obtained by centrifugation at 12,000× *g* for 10 min. To quantify the total phenolic acid content (TPC), the Folin–Ciocalteu reagent and gallic acid were used, following the method described by a previous study [61]. In brief, 400 µL of the methanol extract was mixed in a test tube containing 0.6 mL of double-distilled water and 0.25 mL of 50% Folin–Ciocalteu. Next, 750 µL of 15% Na_2_CO_3_ was added and thoroughly mixed. The mixture was then placed at room temperature in a dark environment for 2 h. After incubation, 200 µL of the reaction solution was transferred to a microplate for absorbance determination at 760 nm using a microplate reader (SpectraMax M2, Molecular Devices, Sunnyvale, CA, USA). For the determination of the total flavonoid content (TFC) in the fruit extracts, aluminum chloride reagent was used. In summary, 1.5 mL of 80% methanol, 0.1 mL of 10% aluminum chloride hexahydrate, 0.1 mL of 1 M sodium acetate, and 2.8 mL of deionized water were combined with 0.5 mL of the extract solution. After 40 min, the mixture’s absorption was measured at a wavelength of 415 nm to determine the control’s absorbance.

### 5.3. Metabolomics Profiles

Widely targeted and targeted metabolomic profiling were performed at MetWare Biotechnology Limited Company (Wuhan, China). Extraction and metabolite analyses were performed as described by Yang et al. [33]. For widely targeted metabolomic analysis, the sample extracts were analyzed using an LC-ESI-MS/MS system (UPLC, Shim-pack UFLC SHIMADZU CBM A system, https://www.shimadzu.com/, accessed on 22 May 2022; MS, QTRAP^®^ 4500+ System, https://sciex.com/, accessed on 22 May 2022). Metabolites with fold change ≥1.5 or ≤0.67 were defined as DAMs. Anthocyanin contents were detected based on the AB Sciex QTRAP 6500 LC-MS/MS platform. The sample was freeze-dried, ground into powder (30 Hz, 1.5 min), and stored at −80 °C until needed. A mass of 50 mg powder was weighed and extracted with 0.5 mL of methanol/water/hydrochloric acid (500:500:1, *v*/*v*/*v*). The sample extracts were analyzed using a UPLC-ESI-MS/MS system (UPLC, ExionLC™ AD, https://sciex.com.cn/, accessed on 1 January 2023; MS, Applied Biosystems 6500 Triple Quadrupole, https://sciex.com.cn/, accessed on 1 January 2023). Metabolites with fold change ≥2 or ≤0.5 and VIP (variable importance in project) ≥ 1 were defined as DAMs. Citrus lipid contents were detected by MetWare (http://www.metware.cn/, accessed on 6 March 2023) based on the AB Sciex QTRAP 6500 LC-MS/MS platform. Extraction of lipids was carried out using a solvent system of methanol, methyl tert-butylether (MTBE). The sample extracts were analyzed using a UPLC-ESI-MS/MS system (UPLC, ExionLC™ AD, https://sciex.com.cn/, accessed on 12 April 2023; MS, Applied Biosystems 6500 Triple Quadrupole, https://sciex.com.cn/, accessed on 12 April 2023). Metabolites with |Log2 Fold-change| ≥ 1 and VIP ≥ 1 were defined as DAMs.

### 5.4. Determination of Antioxidant Activity

To assess the antioxidant activity of the citrus fruit samples, we employed biochemical methods, specifically the FRAP, ABTS, and DPPH radical scavenging assays, each conducted in triplicate. For the ferric reducing antioxidant power (FRAP) assay, the method by Benzie et al. [62] was followed with slight modifications. Three aqueous stock solutions containing 0.1 M acetate buffer (pH 3.6), 10 mM TPTZ [2,4,6-tris(2-pyridyl)-1,3,5-triazine] in 40 mM hydrochloric acid solution, and 20 mM ferric chloride were prepared and stored under dark conditions at 4 °C. Prior to analysis, these stock solutions (in a 10:1:1, *v*/*v*/*v* ratio) were combined to create the FRAP reagent. Aliquots of 50 μL of the sample extracts were mixed with 1.5 mL of freshly prepared daily FRAP working reagent. The absorbance was measured after 30 min at 593 nm.

The scavenging activity of ABTS radicals (ABTS+) was elevated using the method by Zulueta et al. [63] with slight adjustments. Briefly, 50 μL aliquots of the sample solutions were mixed with 1.0 mL of the ABTS•+ working solution. Each mixture was incubated in the dark at 25 °C for 5 min, and the absorbance was measured at 734 nm.

For the DPPH radical scavenging assay, we followed a previously reported method [64]. In summary, 160 μL of 0.15 mM DPPH solution was mixed with 40 μL of a sample solution and incubated at room temperature for 30 min, after which the absorbance at 517 nm was evaluated.

### 5.5. Prediction of the Potential Active Ingredient

All secondary metabolites identified in metabolomics were subjected to the TCMSP (Traditional Chinese Medicine Systems Pharmacology Database and Analysis Platform, https://www.tcmsp-e.com/, accession data on 25 April 2024) to find potential active ingredients [56]. In accordance with the drug screening criteria (OB ≥ 20%, DL ≥ 0.1) suggested by the TCMSP database, metabolites that met these requirements were considered as potential active ingredients. Target genes of the potential active ingredient (Probability > 0.1) were searched from the Swiss TargetPrediction (http://www.swisstargetprediction.ch/, accession data on 29 April 2024) and PubChem (https://pubchem.ncbi.nlm.nih.gov/, accession data on 2 May 2024) [65,66].

### 5.6. Prediction of Core Targets of Citrus and Diseases

The term “oxidative damage” was used as the keyword to retrieve disease-related genes from databases such as OMIM (https:/www.omim.org/, accession data on 8 May 2024), DisGeNET (https:/www.disgenet.org/, accession data on 9 May 2024), and GeneCards (https:/www.genecards.org/, accession data on 10 May 2024) [67,68,69]. These common targets were added to the STRING 11.5 database (https://cn.string-db.org/, accession data on 28 May 2024) [70]. Subsequently, Cytoscape 3.9.1 software was utilized to screen the core targets: targets with degree, closeness, and betweenness values larger than their average values were picked as core targets [71].

### 5.7. Gene Ontology (GO) and Kyoto Encyclopedia of Genes and Genomes (KEGG) Analysis

To elucidate the functions and metabolic pathways of the core genes, we imported them into the David database (https://david.ncifcrf.gov/, accession data on 28 May 2024) for GO function (biological process (BPs), cellular component (CCs), molecular function (MF)) and KEGG analysis [72]. Subsequently, the results of the GO and KEGG analyses were visualized and further scrutinized using the bioinformatics website (https://www.bioinformatics.com.cn/, accession data on 8 June 2024).

### 5.8. Statistical Analysis

All experiments were conducted in triplicate, and the data are presented as the mean ± standard deviation (SD). The differences between the mean values of the main factor were determined by a one-way analysis of variance (ANOVA). A post hoc analysis was conducted using Tukey’s test, with 5% statistically significant differences (*p* < 0.05) considered statistically significant. Unsupervised principal component analysis (PCA) and was carried out using R Studio software (2023.06.1-524, https://www.rstudio.com/, accession data on 16 May 2024, R version 4.3.3) with the two packages FactoMineR and factoextra [73]. The column stacking diagram was drawn by R Studio software (R version 4.3.3). The heatmap was drawn by TBtools [74]. The correlation coefficient was determined using Pearson’s correlation coefficient (r).

## Figures and Tables

**Table 1:** The effect of hetero-grafting (Z, ZC, H, X) on the fruit flavor, nutritional quality, and antioxidant capacity.

	TSS (%)	TA (%)	VC (mg/100 mL)	TSS/TA	SC (mg/g)	GC (mg/g)	FC (mg/g)
Z	11.63 ± 0.32 a **	0.98 ± 0.04 a	59.81 ± 1.14 a	11.91 ± 0.56 ab **	23.94 ± 1.32 ab *	19.899 ± 0.95 a **	13.994 ± 0.872 b **
ZC	11.23 ± 0.12 a **	0.85 ± 0.06 a	59.68 ± 0.67 a	13.34 ± 0.73 a **	25.37 ± 1.81 a *	21.776 ± 0.75 a **	16.185 ± 1.138 a **
H	10.5 ± 0.15 b **	0.95 ± 0.03 a	61.72 ± 1.74 a	11.05 ± 0.21 b **	21.64 ± 1.58 b *	17.215 ± 1.45 b **	13.866 ± 1.061 b **
X	10.07 ± 0.15 b **	1.00 ± 0.06 a	63.49 ± 1.66 a	10.09 ± 0.63 b **	21.42 ± 1.04 b *	16.543 ± 1.13 b **	12.517 ± 0.479 b **
	**TAA (mg/g)**	**TPC (mg/g)**	**TFC (mg/g)**	**TAC (mg/g)**	**FRAP (μmol TE/g FW)**	**ABTS (μmol TE/g FW)**	**DPPH (%)**
Z	9.92 ± 0.174 a	0.43 ± 0.02 a **	0.09 ± 0.00 b **	9.17 ± 0.06 a **	6.100 ± 0.138 a **	2.272 ± 0.092 a *	22.396 ± 1.170 a **
ZC	10.96 ± 0.75 a	0.46 ± 0.04 a **	0.10 ± 0.00 a **	8.79 ± 0.37 a **	6.381 ± 0.503 a **	2.298 ± 0.161 a *	23.795 ± 1.453 a **
H	12.08 ± 1.08 a	0.33 ± 0.02 b **	0.08 ± 0.00 c **	4.93 ± 0.24 b **	5.279 ± 0.234 b **	1.762 ± 0.042 b *	17.784 ± 0.684 b **
X	11.12 ± 1.63 a	0.32 ± 0.03 b **	0.08 ± 0.01 c **	3.67 ± 0.14 c **	5.755 ± 0.193 b **	1.761 ± 0.100 b *	15.896 ± 0.698 b **

Different lowercase letters after numbers in the same column represent the difference was significant. * *p* < 0.05 level, ** *p* < 0.01 level. The same as below. TSS: total soluble solids; TA: titratable acids, Vc: vitamin C; GC: glucose; SC: sucrose; FC: fructose; TAA: total amino acids; TPC: total phenolic acid content; TAC: total anthocyanins; ABTS: 2,2′-azinobis (3-ethylbenzothiazoline)-6-sulfonic acid; FRAP: ferric reducing antioxidant power; DDPH: 1,1-diphenyl-2-picrylhydrazyl; TFC: total flavonoid content.

## Data Availability

The raw data supporting the conclusions of this article will be made available by the authors on request.

## Supplementary Materials

The following supporting information can be downloaded at https://www.mdpi.com/article/10.3390/plants13162259/s1.
