# Supplementary material for: Combined Metabolomics and Network Pharmacology Analysis Reveal the Effect of Rootstocks on Anthocyanins, Lipids, and Potential Pharmacological Ingredients of Tarroco Blood Orange (Citrus sinensis L. Osbeck)
Source: Plants (Basel). 2024 Aug 14;13(16):2259. doi: 10.3390/plants13162259 (PMC11358934; doi:10.3390/plants13162259)
Supplement: Supplementary file 1 [file plants-13-02259-s001.zip › supplementary materials.pdf]

Supplementary materials

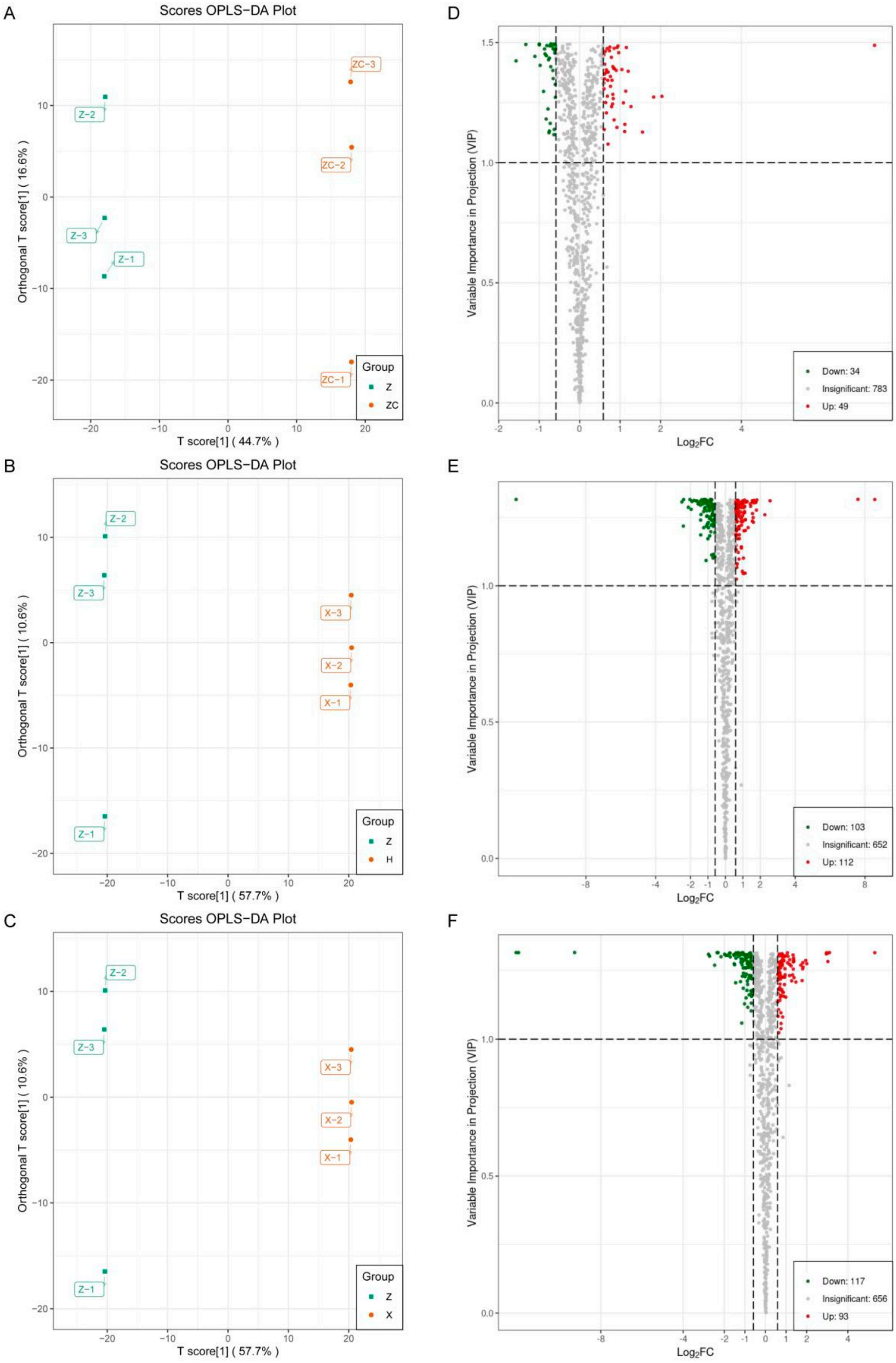

Figure S1. (A-C) The score plots of OPLS-DA pairwise comparisons of differential metabolites. (A), Z vs. ZC; (B), Z vs. H; (C), Z vs. X. (D-F) Volcano plots showing the differential metabolites expression levels. (D), Z vs. ZC; (E), Z vs. H; (F), Z vs. X.

On the left side of vs. is the control group.

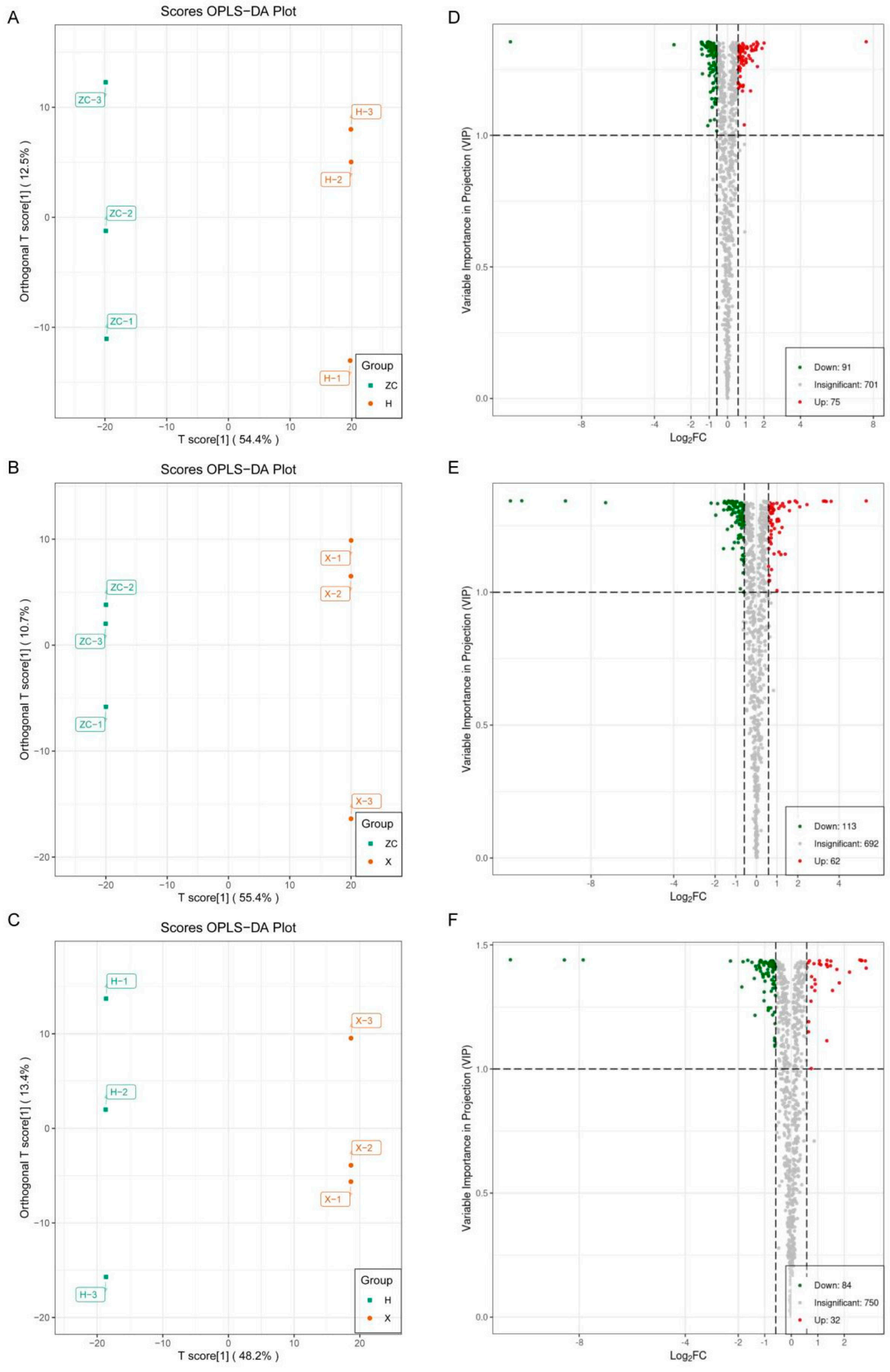

Figure S2. (A-C) The score plots of OPLS-DA pairwise comparisons of differential metabolites. (A), ZC vs. H; (B), ZC vs. X; (C), H vs. X. (D-F) Volcano plots showing

the differential metabolites expression levels. (D), ZC vs. H; (E), ZC vs. X; (F), H vs. X. On the left side of vs. is the control group.

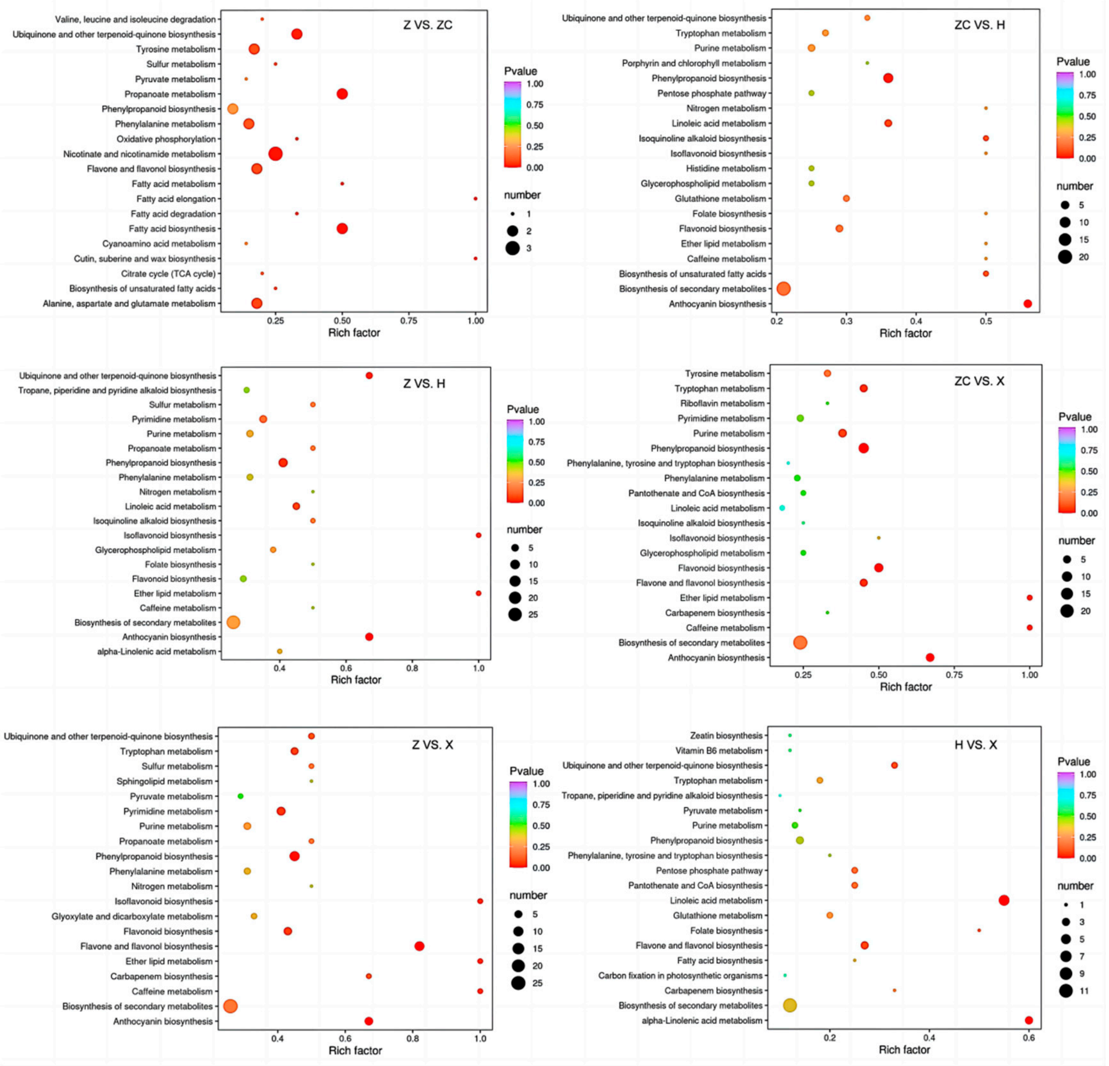

Figure S3. KEGG enrichment analysis of differential metabolites for six comparison groups (Z VS. ZC, Z VS. H, Z VS. X, ZC VS. H, ZC VS. X, H VS.X). On the left side of vs. is the control group.

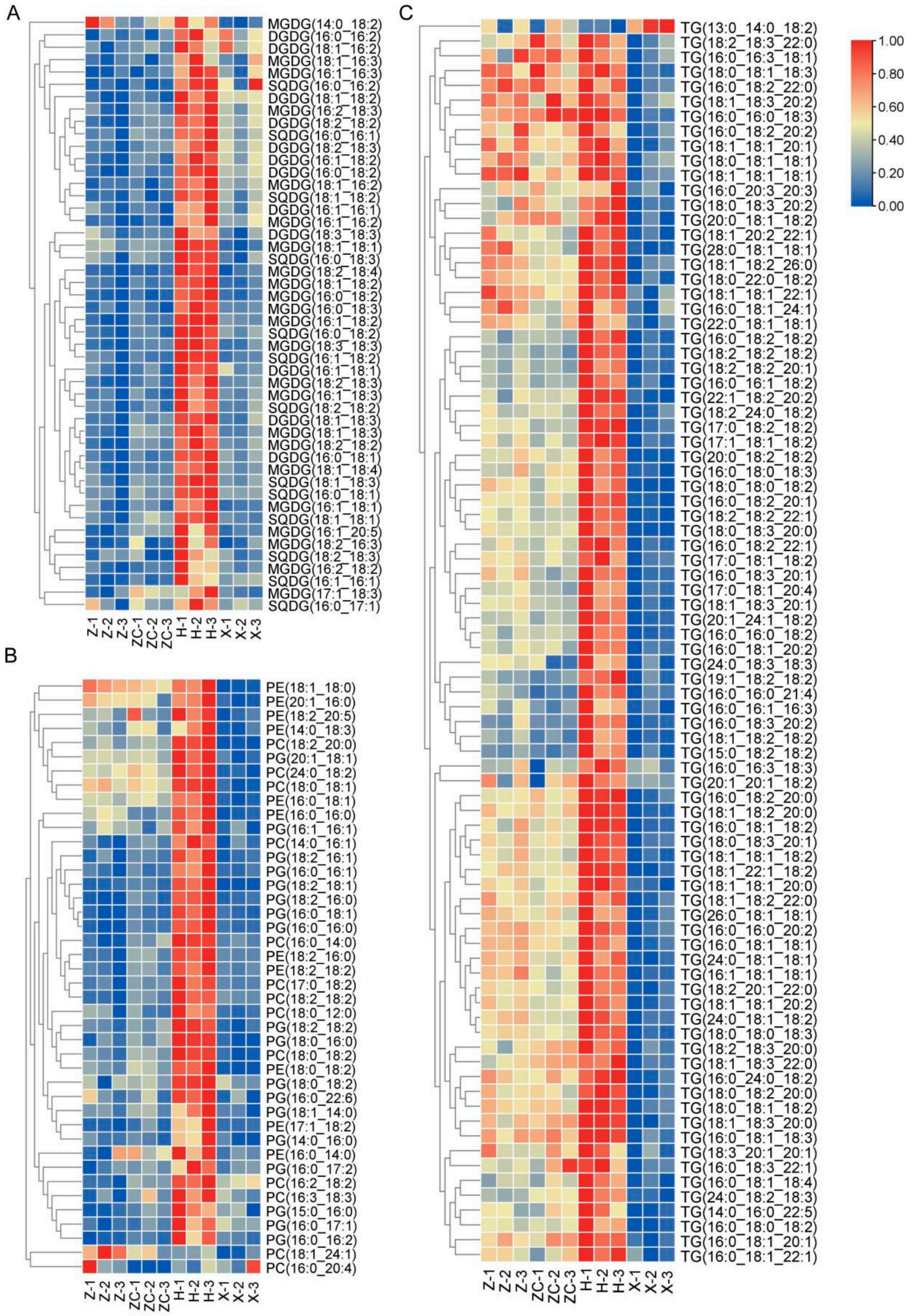

Figure S4. Heatmap of DAMs concentration from lipidomics data. (A) The concentration of glucosylsphingoshine; (B) The concentration of PC, PE, and PG; (C) The concentration of TG.

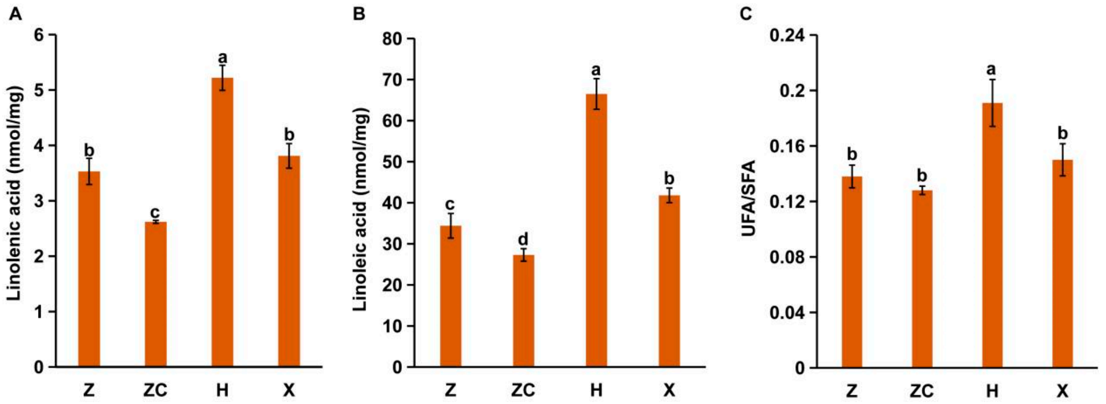

Figure S5. Changes in linoleic acid (A), linolenic acids (B), and UFA/SFA (C) of Tarocco blood orange on four rootstocks. Different letters above the bars on columns show a significant difference at $P < 0.05$ according to the LSD test.

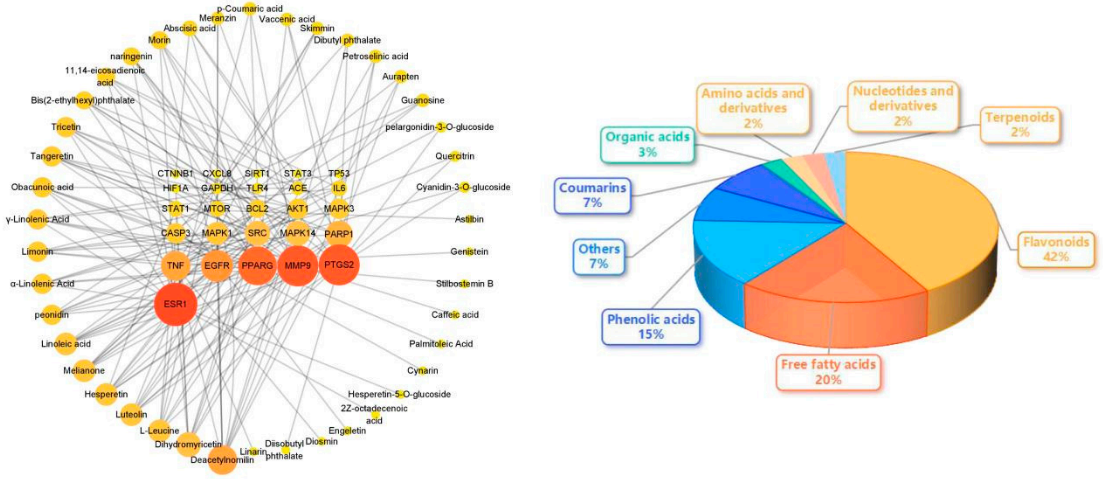

Figure S6. (A) core metabolites-target network. (B) Classification of the 42 core metabolites.
